# Eco‐evolutionary origins and diversification in a megadiverse hotspot: Arthropods in the Greater Cape Floristic Region

**DOI:** 10.1002/ece3.70195

**Published:** 2024-08-16

**Authors:** Michael J. Samways, James S. Pryke, René Gaigher, Charl Deacon

**Affiliations:** ^1^ Department of Conservation Ecology & Entomology Stellenbosch University Stellenbosch South Africa

**Keywords:** adaptation, biodiversity, biogeography, deep time, historic evolution invertebrates, insects, South Africa

## Abstract

The Greater Cape Floristic Region at the southern tip of Africa is a global megadiversity hotspot. The region's biodiversity has been driven by a long history of topographic, climatic, and sea level change coupled with geological uplift, and without being exposed to any major climate events such as glaciations since the breakup of Gondwana. Among arthropods, this long history has led to the survival of many ancient lineages, manifested by much disparity followed by considerable speciation in more recent times, with the emergence of many cryptic species flocks. There is much convergence among the various taxa and functional groups in how they have responded to the various environmental filters of the past. There has also been the development of a great many morphological, behavioral, and microhabitat specialisms, associated with both topography and particular habitats, as well as interactions with other organisms. Morphological and molecular advances are elucidating how this megadiversity came about. There are indications that among the arthropod fauna, especially species that are small‐sized and have cryptic lifestyles, many more taxa remain to be discovered. Here, we review the eco‐evolutionary trends that have occurred in this region and that have resulted in such remarkable arthropod diversity. Conservation of the arthropod fauna requires recognition of this historical biogeography and ecology. Instigation of approaches over wide areas is required so as to encompass all this diversity.

## Graphical abstract

The Greater Cape Floristic Region at the southern tip of Africa is a global megadiversity hotspot. The region's biodiversity has been driven by a long history of topographic, climatic, and sea level change coupled with geological uplift, and without being exposed to any major climate events such as glaciations since the breakup of Gondwana. Here, we review the eco‐evolutionary trends that have occurred in this region and that have resulted in the remarkable arthropod diversity.

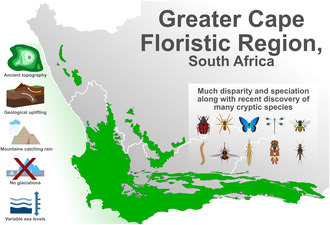

## 1 INTRODUCTION

**FIGURE 1:**
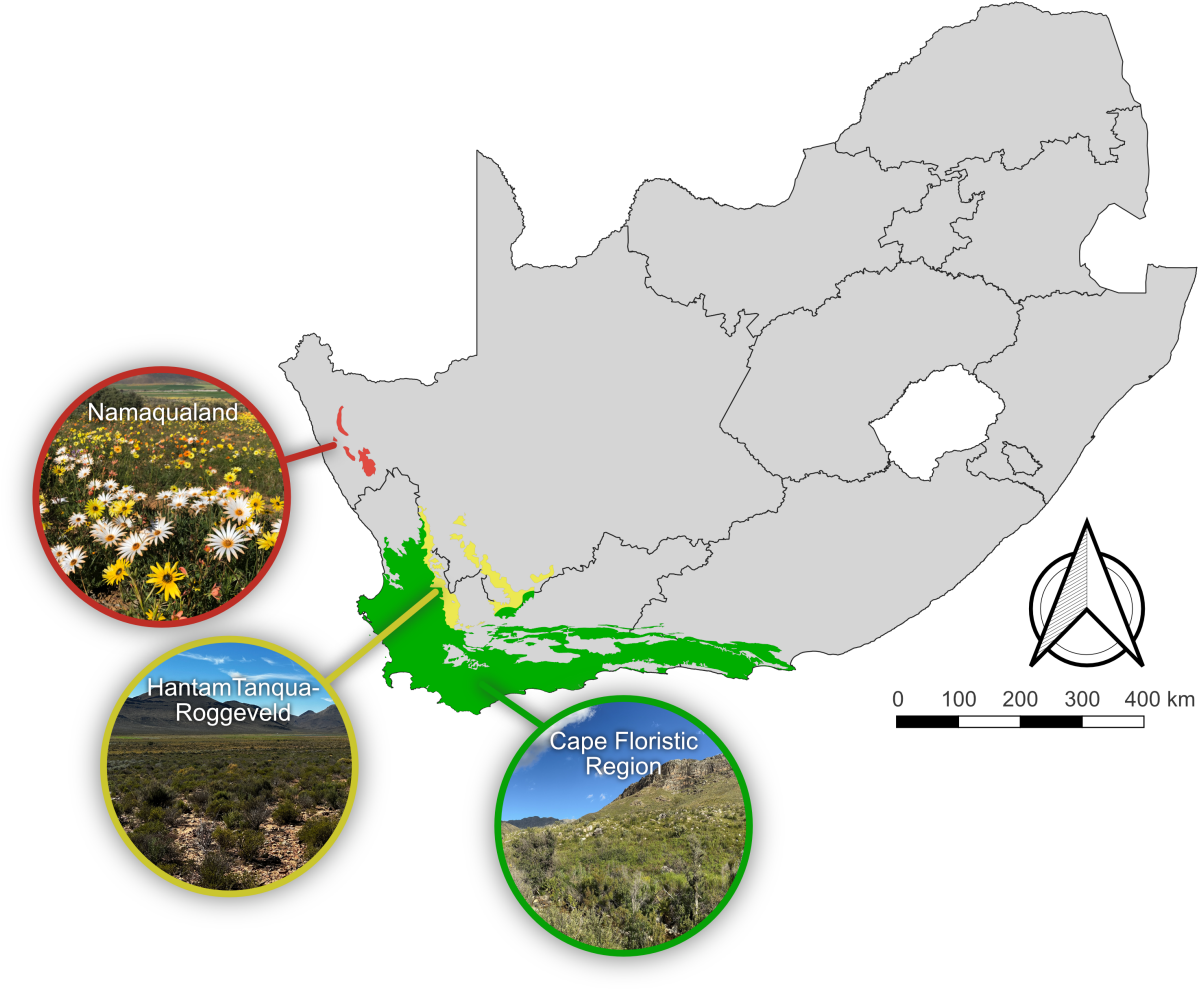
The Greater Cape Floristic Region (GCFR) is at the southwestern tip of South Africa. It includes the Cape Floristic Region, the HantamTanqua‐Roggeveld Region, and the Namaqualand Region.

The Greater Cape Floristic Region (GCFR) includes areas that are floristically distinct from the rest of southern Africa, and is composed of the Cape Floristic Region, the HantamTanqua‐Roggeveld Region, and the Namaqualand Region (Figure 1). The GCFR is well‐known for its high levels of floristic diversity and endemism covering a broad range of climatic conditions from mesic to arid (Born et al., 2007). Significantly, the GCFR is topographically highly variable, and an important driver of the whole region's ecosystems. In the Cape Floristic Region in the southwest of the GCFR, the mountains act as water sponges soaking up rainfall and condensation during the cool, wet winters while gradually releasing water into streams and small rivers during the hot, dry summers, many of which are perennial (Bradshaw & Cowling, 2014). However, rainfall seasonality evens out eastwards, with some summer rains in the far eastern part (De Moor & Day, 2013).

Although GCFR plant diversity is reasonably well known (Cowling et al., 1998; Desmet, 2007; Goldblatt & Manning, 2002), relatively little is known of the comparative levels of diversity or GCFR endemism among the arthropods, especially for the more inconspicuous groups. This is because biodiversity levels are extremely high, with much more exploration and research still required to describe all the species. Furthermore, there appear to be many sets of cryptic species as revealed by molecular analyses (Myburgh et al., 2021). Nevertheless, some key points are emerging, all stemming from a long but varied palaeo‐history.

The objectives here are principally to explore and synthesize the eco‐evolutionary arthropod findings to date, which has yet to be done. We performed a literature search on the Web of Science online database, searching article titles, abstracts and keywords using the terms: (“cape floristic region*” OR “cape floral kingdom*” OR “namaqualand*” OR “namakwaland*” OR “hantamtanqua*” OR “hantamtankwa*” OR “hantam‐tanqua*” OR “hantam‐tankwa*” OR “cape province*” OR “western cape*” OR “cape fold mountain*” OR “cape fold belt*” OR “hottentots‐holland*” OR “hottentots holland*” OR “cederberg*” OR “cedarberg*” OR “swartberg*” OR “table mountain*”) AND (“ecoevol*” OR “eco‐evol*” OR “endemi*” OR “phylogeo*” OR “evol*” OR “biogeo*”) AND (“arthropod*” OR “insect*” OR “invert*” OR “macroinvert*” OR “macro‐invert*” OR “coleopt*” OR “beetle*” OR “hemipt*” OR “bug*” OR “dipt*” OR “fly*” OR “odonat*” OR “dragonfl*” OR “damselfl*” OR “hymenopt*” OR “wasp*” OR “bee*” OR “ant*” OR “arach*” OR “arane*” OR “spider*” OR “ephemeropt*” OR “mayfl*” OR “plecopt*” OR “stonefl*” OR “trichopt*” OR “caddisfl*” OR “manto*” OR “mantis*” OR “orthopt*” OR “grasshopper*” OR “katydid*” OR “cricket*” OR “lepidopt*” OR “butterfl*” OR “moth*” OR “myriapod*” OR “milliped*” OR “chilopod*” OR “centiped*” OR “collemb*” OR “springtail*” OR “neuropt*” OR “lacewing*” OR “scorpion*”). The search resulted in 622 articles, each of which was manually screened for inclusion in this review. Articles from outside the GCFR, those that did not investigate the evolutionary history of arthropods specifically, and those that investigated more recent impacts such as habitat transformation, contemporary climate, and so on, were excluded from the review. Relevant references within considered articles were also included.

Articles were studied in detail and categorized into a set of topics. Firstly, we focus on the importance of geomorphology and palaeo‐environments in shaping current plant diversity and patterning as a driver of significant components of present‐day arthropod diversity. This leads to the discussion of current known levels of arthropod diversity and endemism, both terrestrial and aquatic. Then follows the discussion of plant–arthropod relationships, especially pollination and levels of congruency between the two groups. Two major important contemporary drivers, fire and El Niño Southern Oscillation events, are then explored. We then focus specifically on the Cape Peninsula which has exceptionally high levels of endemism among many arthropod groups. Finally, we discuss how this extensive background might direct conservation efforts in the region.

## 2 GEOMORPHOLOGY AND PAST CLIMATES

**FIGURE 2:**
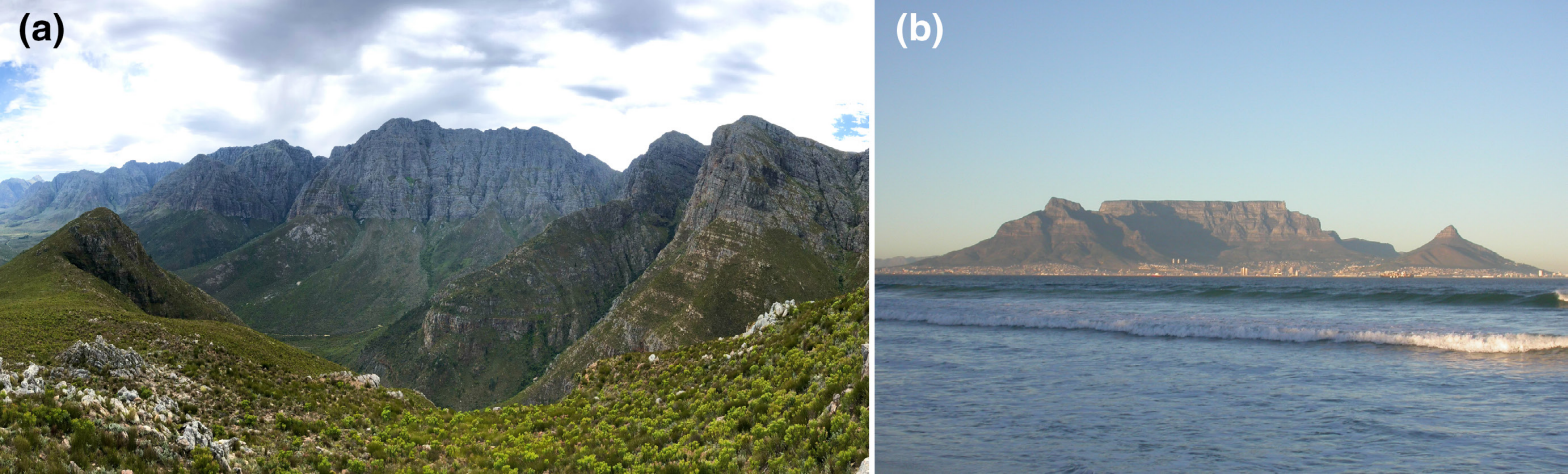
The ancient Cape Fold Mountains (a) have a strong influence on regional climate gradients. Table Mountain (b) is an inselberg, today surrounded by the City of Cape Town.

A significant geomorphological feature of the GCFR is the ancient Cape Fold Mountains, which dominate the southern rim of the region and have had, and continue to have, a major effect on the biota (Figure 2). The coarse granite bedrock, laid down about 540 mya, is overlain mostly by hard quarzitic sandstone sedimentary rock deposited after that time until about 300 mya. Around 250 mya, there were considerable crust movements, leading to geological folding throughout much of the Cape Fold Mountains. One notable exception is the iconic Table Mountain, Cape Town, which remained layered as an inselberg (Norman & Whitfield, 2006) (Figure 2).

The Mesozoic Era (252–66 mya) saw the break‐up of Pangaea, with major consequences for the palaeocontinental configuration, palaeoclimates, and biological evolution. Cool and warm conditions alternated, with even some hothouse conditions, when the global average temperatures were around 6–9°C warmer than today. There was also extensive evaporation as well as desert conditions. Global sea levels were mainly high, and atmospheric O_2_ varied between 15% and 25%. Degassing from volcanism linked to the rifting process of Pangaea and methane emissions from reptilian biotas were climate‐influencing factors that enhanced atmospheric CO_2_ concentrations up to 16 times compared to today's levels. Continental break‐up modified landmass palaeopositions and shoreline configurations, resulting in large oceans and greatly altering oceanic circulation. There were also important meteorite impact events, with at least nine huge (diameter >20 km) impact structures known (Holz, 2015).

The Cenozoic Era (66 mya‐present) began following the massive meteorite impact that ended the Mesozoic. The first Period in the Cenozoic was the Paleogene (66–23 mya). It began with the recovery of biological communities in a warmer environment than today. The Paleogene is also characterized by much tectonic activity, volcanism, orogeny, and climate shifts, with high levels of precipitation and erosion. Temperatures in the late Paleocene‐early Eocene (about 57–55 mya) (the Paleocene‐Eocene Thermal Maximum; PETM) peaked at several degrees higher than today, followed by much cooling during the Eocene–Oligocene transition (33.9 mya). In the GCFR, there was then a tropical‐subtropical climate until the middle Miocene (about 14 mya), followed by a drier climate for the next 3 million years or so. Then from about 10 to 8 mya, a more seasonal climate of cool, wet winters and hot, dry summers prevailed in the southwest, remaining to this day (Linder & Hardy, 2004). In the last 3.5 my, rainfall patterns have likely been relatively constant (Maslin et al., 2012). After the last ice age around 12,000 years ago, the El Niño Southern Oscillation (ENSO) arose resulting in distinct wet and dry cycles (Gagan, 2009).

## 3 EFFECTS OF PAST CLIMATES AND SEA LEVEL CHANGES ON THE FLORA

### 3.1 Deep time climates and the flora

The climatic swings in the Paleogene had a profound effect on plant communities and varied according to latitude, elevation, and degree of continentality (Korasidis et al., 2022). These temperature fluctuations were also accompanied by changes in sea levels, hydrological patterns, and extreme weather events, including at southern latitudes (Sluijs et al., 2011).

Significant plant radiations occurred throughout the Oligocene (33.9–23 mya) and Miocene (23–5.3 mya, the first Epoch of the Neogene (23–2.6 mya)), with net plant diversification rates in the GCFR having remained constant through time at globally moderate rates. Soil type shifts were the most important cause of speciation in the genera *Babiana*, *Moraea*, and *Protea*, while shifts in fire‐survival strategy were the most important factor for Podalyrieae. In other plant groups, such as orchids, pollination syndromes displayed much phylogenetic conservatism, including groups with many specialized pollination syndromes, like *Moraea*. It appears that a combination of complex environmental conditions, together with relative climatic stability, promoted high speciation and together with low extinction rates are the most likely reasons for current patterns of megadiversity in the GCFR (Schnitzler et al., 2011). Overall, the evidence suggests that current plant diversity in the region is the product of recruiting diverse lineages over the entire Cenozoic, followed by localized diversification which increased species richness of at least some of the lineages over a long period in an environmentally heterogeneous area (Linder, 2005).

GCFR plant clades show Austral rather than African relationships, although other patterns suggest a cosmopolitan flora, with no simple explanation that accounts for the present‐day distinctive GCFR flora. This flora has been assembled over a long time, from about 80 mya (Galley & Linder, 2006). However, there are palaeo‐endemic and neo‐endemic lineages, with current species richness within this flora the result of several radiations, some of which started in the middle Tertiary (Eocene–Oligocene), followed by much speciation among some clades occurring more recently. The persistence of these various lineages has been possible through the lack of major extinction events, as happened for example during the Quaternary glaciations in Europe. In sum, present‐day floral diversity is the cumulative effect of many lineages radiating, perhaps more rapidly than in other areas, over a long period of time, and likely also the result of much topographic and environmental heterogeneity (Linder, 2005).

However, there are differences in the biomes of the GCFR. Succulent karoo‐endemic lineages are <17.5 my, and most <10 my, suggesting that this biome is the result of recent radiation, probably triggered by climatic deterioration since the late Miocene. In contrast, fynbos‐endemic lineages show a broader age distribution, with some lineages originating in the Oligocene, but most being more recent. These patterns reflect the greater age of fynbos lineages along with considerable recent speciation, probably through a combination of climatically induced refugium fragmentation and adaptive radiation (Verboom et al., 2009).

### 3.2 Sea level change and the flora

During the Neogene, sea levels have fluctuated considerably over the last 22 my due especially to tectonic uplift events (Cowling et al., 2009; Maslin et al., 2012). In the past 3 my, global temperatures have fluctuated, with alternating ice accumulation followed by melting in mostly the northern hemisphere (Miller et al., 2020). Sea transgressions and recessions resulted in sea levels varying between 130 m below and 10 m above current levels (Cowling et al., 2009; Miller et al., 2020).

During the last Pleistocene Glacial Maximum, the southern Palaeo‐Agulhas Plain was composed of highly productive ecosystems (Marean et al., 2020) which extended the GCFR much farther southwards. Today, this extended area is mostly submerged by the Indian‐Atlantic Ocean interface. The sea level changes led to deposition of calcareous sandy substrata along the coast during the last 2.5 my, and along with erosion cycles, have changed soil composition and increased its heterogeneity (Cowling et al., 2009; Hoffmann et al., 2015; Verboom et al., 2015). The current coastal plain has a strong pH gradient associated with the calcareous substrata. These, together with soil depth and texture, have acted as important edaphic filters that led to the incorporation of lineages from floras on juxtaposed substrata, making the area extremely rich in plant species (Grobler & Cowling, 2021).

### 3.3 Spatial variation in the flora

Within the GCFR, plant diversity is richer in the west, where there is also a significant effect of topography, coinciding with the transition from reliable winter rainfall in the west to less reliable non‐seasonal rainfall in the east. Interestingly, rare plant diversity mirrors that of overall plant diversity. It appears that regional plant diversity patterns are the product of different speciation and extinction histories, leading to different steady‐state diversities. Relatively greater stability of the Pleistocene climate in the west would have led to higher speciation rates and lower extinction rates than in the east where Pleistocene climates would largely have not favored GCFR lineages. Possibly also, a more predictable western seasonal rainfall would have favored non‐sprouting plants, resulting in higher speciation and lower extinction rates. Both perspectives are consistent with the higher incidence of rare species in the west, where there are higher levels of beta and gamma diversity associated with environmental and geographical gradients. As rare species contribute little to community patterns, biological heterogeneity is uniform across the GCFR (Cowling & Lombard, 2002).

### 3.4 Plant diversity patterns

The effect of past environmental drivers combined with great topographic heterogeneity has more recently been confirmed (Procheş et al., 2009; van Santen & Linder, 2020; Verboom et al., 2015), as well as the importance of relatively constant seasonal rainfall patterns (Potts et al., 2013) and relatively poor dispersal abilities among plants (Dynesius & Jansson, 2000; Linder, 2003) which likely promoted much plant diversity in the region. Similarly, in tropical East Africa, mountains have also played a vital role as both generators and maintainers of high levels of local plant biodiversity (Dagallier et al., 2020).

Furthermore, the southwestern Cape Floristic Region maintains almost twice as many plant species as the southeastern part, with a distinct west–east plant diversity gradient (Tolley et al., 2014). Drivers of this distribution pattern and high diversity are various, including relatively stable Pleistocene climatic conditions in montane regions, a west–east climatic gradient caused by the cold Benguela Current affecting rainfall and condensation patterns, and repeated ocean transgressions and regressions, all of which have affected the lowlands and have created a mosaic of heterogenous soil types (Linder & Verboom, 2015).

## 4 TERRESTRIAL ARTHROPOD DIVERSITY AND ENDEMISM

Besides high levels of diversity and endemism among insects and other arthropod taxa in the GCFR, there is also considerable species turnover at various spatial scales, driven by both abiotic and biotic factors. Local insect evolution has been characterized by the survival of ancient clades, much diversification among others, with overall considerable habitat, host, and behavioral specialization among many groups (Scholtz et al., 2021). Abiotic factors such as climate fluctuations in the past, and the effect of the Cape Fold Mountains as a movement barrier, have played a major role in the diversification of the GCFR arthropod fauna (Myburgh & Daniels, 2022).

We now consider various GCFR arthropod taxa that have been investigated with respect to their origins and evolution, both in deep and more recent times and relative to other biogeographical areas where information is available.

### 4.1 Beetles

Beetles are numerous, speciose and have been well‐researched in the GCFR. The charismatic lucanid beetle genus *Colophon* is endemic to the GCFR. This group was split into “plesiomorphic” and “apomorphic” clades based on morphology and consists of 22 described species (Durie et al., 2024; Endrödy‐Younga, 1988; Jacobs et al., 2015). A study on 12 of the 22 described species to date indicates that the mean estimated genus divergence time was mid‐Cretaceous, with a split into two lineages during the early Paleocene to mid‐Eocene, with one lineage (the apomorphic clade) following the east–west line of the Cape Fold Mountains, with the exception of *C. westwoodi* that occurs at the most south‐westerly point of the *Colophon* distribution, and the other following a rough north–south line (the plesiomorphic clade) (Switala et al., 2014).

**FIGURE 3:**
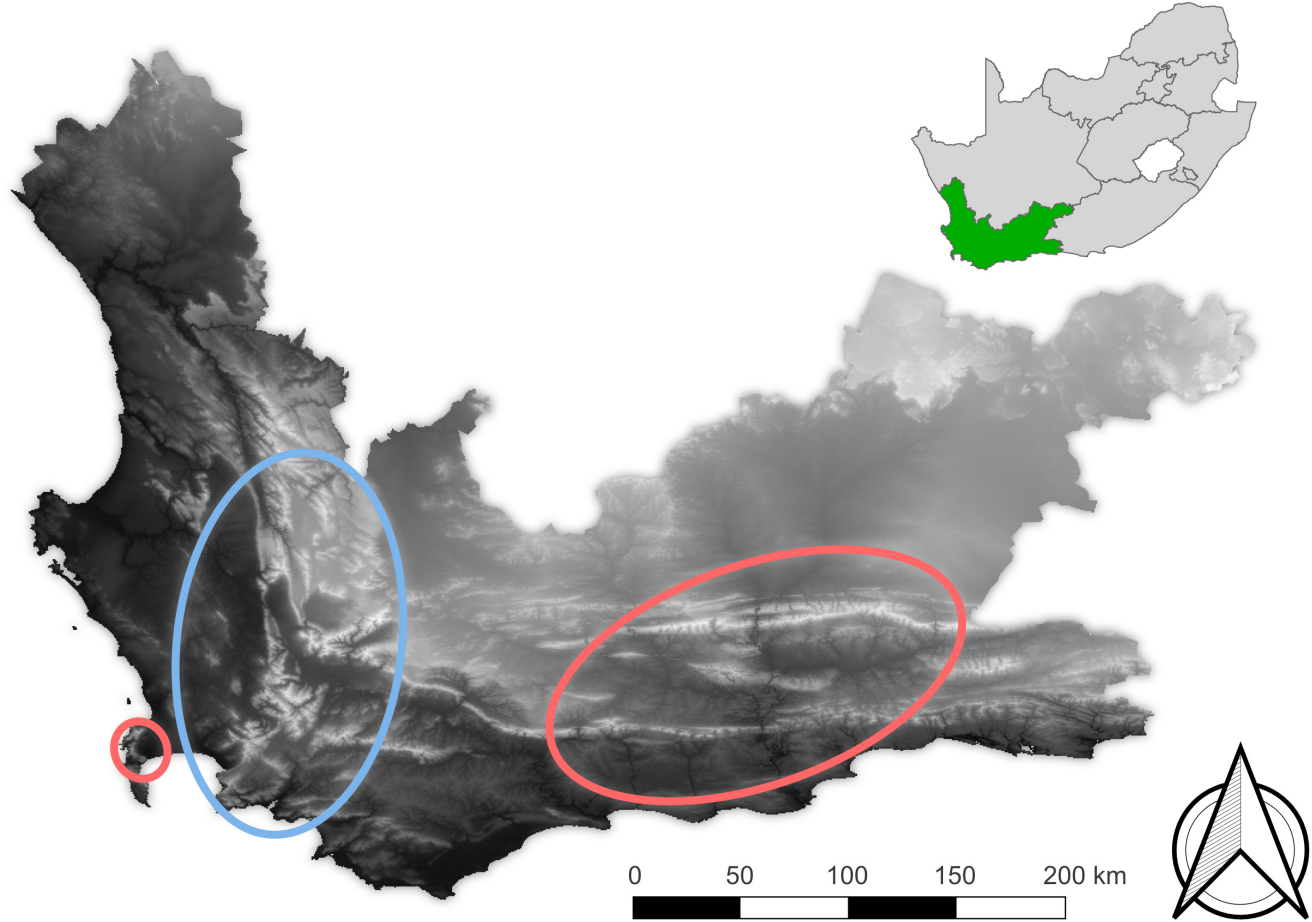
Topography (black: elevation close to sea level; white: high elevation) throughout the Western Cape, South Africa, has had an influence on *Colophon* (Coleoptera: Lucanidae) distribution. Both *Colophon* clades are confined to high‐elevation areas. One clade (blue) today has a north–south line distribution and another clade (red) has an east–west line distribution. The genus appears to have a lowland origin, although today it is confined to the mountains.

Species divergence of these lineages occurred mid to late Miocene, and the genus displays an allopatric distribution. Climate appears to have been the main driver of current *Colophon* distribution, although it is unclear if this is due to the physiological response from the larvae, refugia from modern beetles, or simply where remnant habitat remains. On mountain tops they show preference for moist restionaceous plant beds where the adults can dig into the deeper soils and lay eggs, providing food and protection from fire for the larvae (Roets et al., 2013). Although today *Colophon* species are confined to the mountains, along with the “apomorphic” group despite its name is now considered the older lineage, it now appears they have a lowland origin from the southeastern side of the GCFR (Switala et al., 2014) (Figure 3).

The megadiverse beetle tribe Sericini (Scarabaeidae) has a West Gondwanan origin (about 112 mya), with their vicariance in their early history separating Neotropical and Old World Sericini. However, the subsequent lower Cretaceous biogeography of the group was characterized by repeated migrations out of Africa, resulting in the colonization of Eurasia and Madagascar. North America was colonized from Asia during the Cenozoic and a lineage of more recently evolved Sericini reinvaded Africa. Diversification dynamics suggest three independent shifts to increased speciation rates in African ant‐adapted *Trochalus*, Oriental *Tetraserica*, and Asian and African Sericina. Southern Africa is both the cradle and refuge of the tribe, with the region retaining many old lineages (Eberle et al., 2017).

The weevil (Curculionidae) genera *Rhinusa* and *Gymnetron* show multiple lineages that are disjunct between southern Africa and the Palaearctic. With a southern African origin, the earliest divergence within the group was about 27 mya, with subsequent range expansion into the Palaearctic, likely facilitated by a Miocene “arid corridor” running south–north across Africa. However, vicariance events occurred among southern African and Palaearctic lineages, seemingly due to late Miocene geological and climatic disruption of the arid corridor (Hernández‐Vera et al., 2013).

The flightless beetle tribe Dendarini (Tenebrionidae) is species‐rich in southern Africa, as elsewhere. However, the tribe has a southern African‐Palaearctic disjunction that is ancient in origin and likely due to the progressive fragmentation of the pan‐African rainforest that started in the early Eocene. This, and increased aridification associated with ongoing global cooling that started at that time, strongly influenced the diversification and distribution of these beetles (Kamiński et al., 2022). The flightless *Phoberus capensis* (Trogidae) beetle species complex shows Pliocene‐Pleistocene diversification, driven by climate and the presence of high mountain peaks and sheltered Afrotemperate forest patches acting as important refugia (Strümpher et al., 2016).

Weevils (Curculionoidea) show particularly high diversity and GCFR endemism levels, with thousands of species and many still to be described. A new tribe (Namaini) of the soil‐inhabiting Entiminae now incorporates six new genera (Meregalli et al., 2021). The genus *Brachycerus* alone includes hundreds of species in the GCFR (Oberprieler, 2014). Studies on species in the genus *Phlyctinus* suggest that they started diversifying in the late Miocene, followed by contrasting diversification dynamics for the three inferred clades, with their present disjunct distributions driven by allopatric speciation. Overall, speciation in the genus suggests an interplay between topography and recurring cycles of coastline‐habitat fragmentation resulting from sea level oscillations, inducing diversification of the most speciose clade. In the other two clades, populations likely remained connected, hampering allopatric speciation (Hévin et al., 2022).

In contrast, the Afromontane tropical forest fragments in the GCFR are small, but nevertheless with a canopy beetle species richness that is lower than predicted given the high latitude and the complex and variable climatic conditions of the past. However, like tropical forests, they contain many rare beetle species, many of which are still to be described (Swart et al., 2021).

### 4.2 Butterflies and moths

Two important GCFR lycaenid butterfly genera *Chrysoritis* (with 68 nominate species and sub‐species) and *Thestor* (34 nominate species and subspecies) are attended by specific host ants that feed on exudates of the butterfly larvae (Heath et al., 2023; Heath & Pringle, 2004). Importantly, these butterflies have mostly extremely small geographic ranges. Talavera et al. (2020) recovered monophyletic clades for both *Chrysoritis chrysaor* and *Chrysoritis thysbe* species groups, with the estimated age of divergence for the genus being 32 mya, followed by rapid diversification of the *thysbe* species‐group in the Pleistocene 2 mya. The western fynbos is the most likely region of origin for the radiation of this species group. Colonization of this region occurred 9 mya, followed by a long period of relative stasis before a recent increase in diversification, suggesting that the *thysbe* radiation probably did not arise from colonization of new biogeographic areas. Rather, the impact of species interactions (with mutualistic ants and host plants), the appearance of key behavioral innovations, and/or the opening of new ecological niche space in the region appears to explain the sudden burst of speciation that occurred in the *thysbe* group 2 mya. These findings suggest that there needs to be taxonomic revision of the genus. This is also likely to be the case with many other lycaenid butterflies in the region showing discontinuous distributions across the GCFR.

The butterfly genus *Lepidochrysops* is monophyletic, diverging >15 mya, although the last common ancestor of extant lineages only goes back about 6.4 my, after parasitism of ant associates arose along the 10 my‐long stem branch. *Lepidochrysops* consists of two main clades, with one originating in the southern African montane grasslands and dispersing from there to the GCFR. The other clade originated farther north, and from there spread throughout the woodland and grassland biomes, with a few species reaching the southern African montane grasslands, before a single dispersal to the fynbos. It appears that aridification during the Miocene selected for a phyto‐predaceous (plant feeding as a young caterpillar but parasitic on ants when older) life history style, with ant nests likely providing caterpillars a safe refuge from fire and a source of food when vegetation was scarce (Espeland et al., 2023).

The opening of habitats associated with the emergence of C4 grasslands during the Neogene was a major event in the evolution of biomes, especially in Africa. Noctuid stemborers (Sesamiina) are mostly associated with such open habitats, centred in the Afrotropics. These moths have a Miocene origin in southern and East Africa followed by range expansions associated with the opening of an eastern arid corridor. Speciation events followed, often on highlands that probably acted as climate refugia during cycles of forest fragmentation and reconnection. From the ancestral adaptation to open habitats, maintained over time, transitions to closed habitats were rare and unidirectional. Furthermore, life history and habitat changes led to specific adaptations, such as variations in larval behavior and color. Yet forest‐adapted *Bicyclus* butterflies show almost opposite responses with respect to the opening of habitats in the Afrotropics (Hévin et al., 2024).

### 4.3 Antlions and lacewings

About half the known species of the charismatic Palparini and Palparidiini antlions occur exclusively in southern Africa. They have an ancient origin with the most recent common ancestor of both groups originating in the Late Cretaceous (about 92 mya). Southern Africa appears to have been both their cradle of diversification and a springboard for successive waves of northern dispersals (Hévin et al., 2023).

The Nemopterinae lacewings of southern Africa also have an ancient origin (about 120 mya). However, most genera appear to have diversified during the middle Eocene and into the middle Miocene (about 44–11 mya) with recent rapid divergence of several of the genera occurring during the late Miocene (about 4.5 mya). The timing of the more recent diversification events seems to follow succulent Ruschioideae distribution shifts associated with climatic oscillations, also with adaptations to feeding on flowers in the families Aizoaceae, Asteraceae, and Molluginaceae (Sole et al., 2013). Other taxa also display considerable diversification associated with plants. These include leafhoppers in the genus *Cephalelus*, specializing on species in the diverse family Restionaceae (Augustyn et al., 2013; see Section 4.5).

### 4.4 Grasshoppers and bush crickets

**FIGURE 4:**
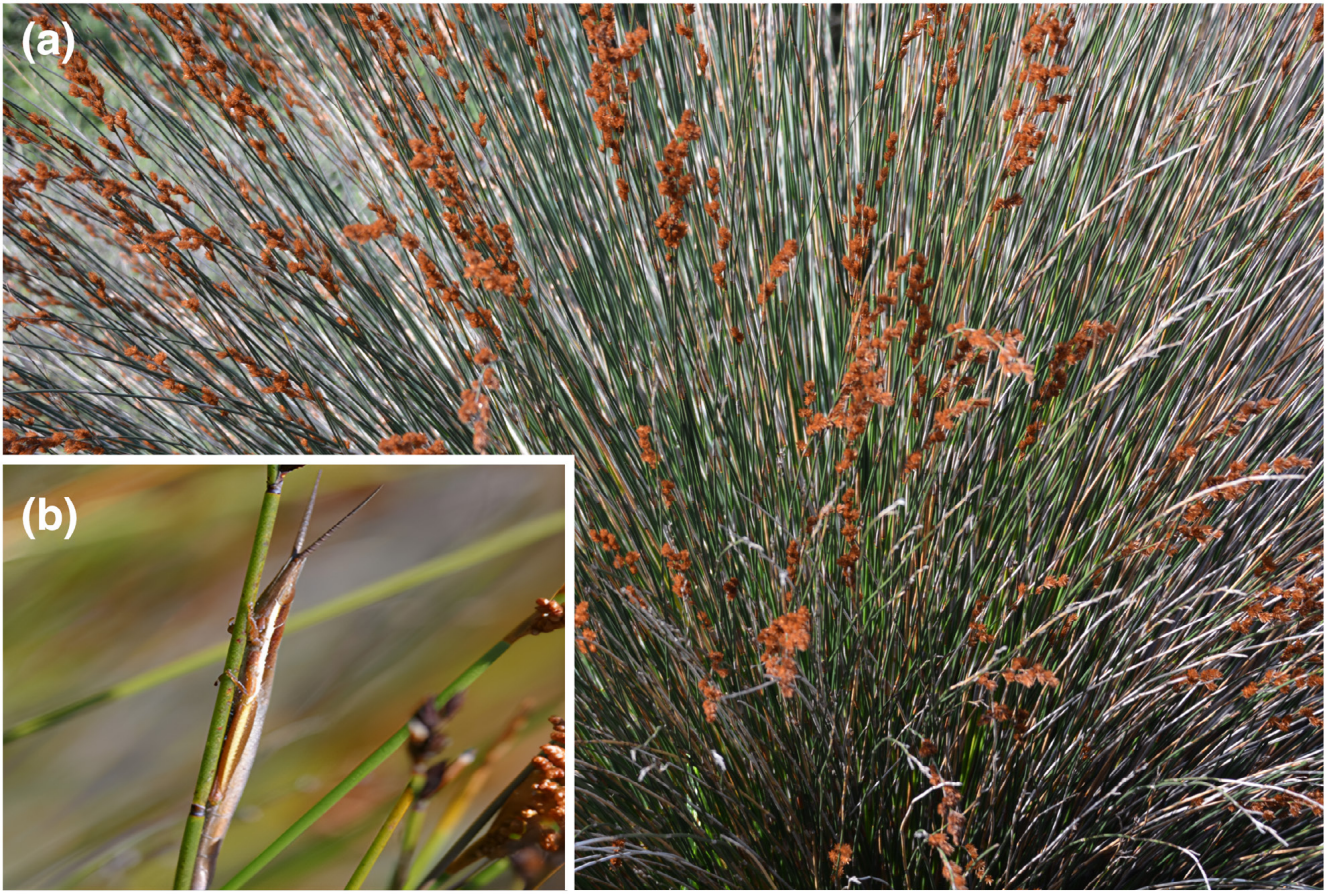
Restionaceae plants occur throughout the Greater Cape Floristic Region (a). *Betiscoides*, a GCFR‐endemic flightless grasshopper genus (b), is strongly associated with Restionaceae plants, resting cryptically on plant stems.

The flightless grasshopper genus *Betiscoides* is endemic to the GCFR and strongly associated with plants of the Restionaceae, where they show strong crypsis adaptation to the plant structure (Figure 4). Molecular evidence suggests that there may be many more species than are currently described. While the five identified main lineages were likely separated during the first phase via dispersal, differentiation occurred later and on smaller spatial scales, mainly driven by vicariance in montane refugia (Matenaar et al., 2018). Interestingly, wind has a strong effect on *Betiscoides* behavior, suggesting that wind, which can often prevail for many days at high velocities, appears to have had an evolutionary effect on these grasshoppers and other species in the GCFR (Matenaar et al., 2014).

Flightless grasshoppers in the genus *Euloryma*, distributed across the GCFR, show much local diversification and morphological adaptation to local conditions based on the description of 21 new species (Spearman, 2013). This again emphasizes the considerable speciation that has taken place in the region and suggests that many unknown species likely remain to be discovered.

South Africa has to date >800 putative species of Orthoptera, with the GCFR identified as an area with the highest orthopteran diversity, species richness, and taxonomic distinctiveness (Gordon et al., 2023). However, bush cricket (Tettigoniidae) alpha diversity in the GCFR is relatively low. The low diversity of this group in the GCFR appears to come from a long history of intense fire and extreme weather events rather than human impact (Thompson et al., 2020), as emphasized by three species of phaneropterine bush crickets surviving well in relatively fire‐free vineyards (Doubell, 2017). While nocturnal predators such as the bat‐eared fox (*Otocyon megalotis*) would seem to have a major impact on the bush crickets in the region, this is likely not the case. Rather, these insects appear to be a low‐return prey resource (Grant & Samways, 2015). Although bush cricket alpha diversity is low, beta and gamma diversity are high, with about 170 putative species to date (Thompson et al., 2017). Bush crickets are normally associated with woody environments, which are scarce in the GCFR. This appears to have led to considerable localized adaptation to low vegetation canopy and windy conditions, as well as much behavioral adaptation to occupying microsites close to the ground in dense vegetation. These adaptations are seen among species in the flightless genus *Brinckiella*, with different species largely associated with specific plant species. Several new species have been discovered, including *Brinckiella aptera* which is completely apterous and so does not sing, the first example of complete aptery in the Phaneropterinae (Naskrecki & Bazelet, 2009).

### 4.5 Cicadas and leafhoppers

The large cicada tribe Platypleurini is of Cenozoic origin (50–32 mya) and widely distributed in Africa and Asia, with generic diversity concentrated in equatorial and southern Africa. African diversification in the speciose genus *Platypleura* and closely related genera coincides with the late Oligocene and Miocene aridification events, which led to forest fragmentation and savanna expansion. Within South Africa, most representatives of *Platypleura* form four distinct clades, each restricted to the separate fynbos, Karoo, forest, and grassland biomes (Price et al., 2019).

The cicada *Platypleura stridula* species complex is restricted to the GCFR. Molecular evidence suggests six clades, each of which has specific host–plant associations and distinct geographical ranges displaying simultaneous radiation events, dating back to the late Pliocene to early Pleistocene, coincident with vegetation change, altered drainage patterns, and accelerated erosion in response to neotectonic crustal uplift and cyclic Pleistocene climate change, as well as global glaciation‐associated changes in climate and sea level (Price et al., 2007). In the case of the *Platypleura plumosa* species complex, river catchments and associated watersheds have driven their diversification, despite these cicadas lacking an aquatic stage (Price et al., 2010).

Restio leafhoppers (tribe Cephalelini) did not co‐diversify with restios (Restionaceae), and diversified much more recently (1–6 mya). However, at the population level, individuals in the *Cephalus* genus are adapted to local conditions by having varying body sizes, body coloration, and host preferences (Augustyn et al., 2017). Variations in trait expressions have a strong relationship with spatial turnover in host plant availability, pointing to host plant availability being an important driver of arthropod diversification in the GCFR.

### 4.6 Scorpions and spiders

Scorpions are an ancient group, first appearing in the Devonian about 381 mya. They diversified prior to the breakup of Pangea, undergoing ancient diversification between the Devonian and early Carboniferous (Sharma et al., 2018). The speciose genus *Parabuthus*, of which there are 15 species in South Africa, exhibits a disjunct distribution between southern Africa and northeastern Africa, as seen in many other taxa, a result of various aridification events from the Pliocene through to the Upper Pleistocene. The center of origin appears to be southern Africa. Range expansion to northeastern Africa appears to have occurred during at least two separate periods of aridity, followed by vicariance during wetter periods, promoting the speciation of scorpion populations isolated in the two regions. Ancestors of *Parabuthus* diverged into unconsolidated sand dune habitats, as well as consolidated sand and sandy‐loam habitats, contemporaneously. The occurrence of reversals in certain species to hard‐surface habitats indicates that this process can evolve in either direction while being relatively plastic, notwithstanding that the major sand systems of southern Africa have likely been significant barriers to range expansion by species preferring hard surfaces in the past (Prendini, 2001).

Endemism among spiders is high, at 60% for all South African species. In turn, the GCFR is a particularly important hotspot of spider endemism (Foord et al., 2020). There are 920 described spider species from the GCFR which is 42% of all species found to date in the whole of South Africa (Dippenaar‐Schoeman et al., 2024).

### 4.7 Velvet worms

The GCFR Onychophora is an interesting group in that the species are confined to Afromontane forests and are composed of many cryptic species. Divergence time estimates indicate that the dominant genus *Peripatopsis* evolved about 60 mya, and the divergence of the main *Peripatopsis balfouri* species complex was about 48 mya. However, within this complex, all the divergence dates fall into the late to early Miocene (<23 mya), while divergence among the three *Peripatopsis capensis* clades were about 21 mya, about 15 mya ago between the two *Peripatopsis sedgwicki* clades, and about 9 mya ago between the two *Peripatopsis clavigera* clades. Areas of endemicity among *Peripatopsis* include the Cape Peninsula, Agulhas Plain (Overberg region), Langeberg region, and the coastal forest areas of the southwestern Cape, suggesting historical isolation of taxa in these regions, largely mirroring plant endemicity patterns (Daniels et al., 2009). With the discovery of several new cryptic species, some inferences are emerging as to how this speciation came about. Climate change was a major speciation driver, beginning with aridification in the late Miocene to late Pliocene. It appears that the ancestor of the *P. clavigera* complex was widely distributed, followed by contraction to forest refugial areas during the dry times. As the climate became wetter again, there was then distributional expansion resulting in the complex pattern of reproductive isolation seen today (Barnes et al., 2020; McDonald & Daniels, 2012).

### 4.8 Millipedes and springtails

Other less mobile arthropod groups, like the millipedes (Diplopoda) also show high levels of local endemism, especially in isolated forest patches (Hamer & Slotow, 2002). In turn, the South African soil biota in general is species‐rich by world standards, with overall both high levels of local endemism and high species turnover. The GCFR ranks highly in species richness and local endemism, especially for Collembola (Janion‐Scheepers, Bengtsson, et al., 2016). The many species in the large collembolan genus *Seira* show pronounced thermal tolerance and desiccation tolerance in the dry fynbos, enabling the various species to exploit and diversify in the region's hot and dry landscapes (Liu et al., 2021).

## 5 AQUATIC ARTHROPOD DIVERSITY AND ENDEMISM

de Moor and Day (2013) overview diversification and local endemism of aquatic biodiversity in the GCFR. Hydrophilic vascular plants show a similarly high rate of endemism to that of terrestrial plants, at 86% endemism. All crustacean species combined are 72% endemic to the region. The rate of endemism in aquatic insects, averaging 54%, ranges from 18% for the Odonata to 92% for Plecoptera. Significantly, taxonomic disparity (significant differences in body form, manifested in higher level taxa) of the GCFR biota is high. In contrast, the terrestrial vegetation displays a very high diversity (many species) but low disparity (relatively few higher taxa, especially genera). This suggests that the driving factor in maintaining a disparate aquatic fauna was the survival of some of the older clades that were formerly widely distributed, while terrestrial plant diversity seems to have resulted from adaptive radiation of few taxa, probably more recently. An example of disparity is seen in the Trichoptera and aquatic Coleoptera and Diptera where familial and generic diversity in the GCFR is particularly high, but less so in Odonata and Plecoptera.

Possible reasons for this high level of endemism are that the ancient Cape Fold Mountains extend through most of the region. Furthermore, the relative geological stability of the region after the break‐up of Gondwana suggests that, apart from various river capture events (resulting in population isolation), river valleys of the GCFR have remained largely unchanged since the Caenozoic (66–65 mya). There were major uplifts about 20 and 5 mya, resulting in the rejuvenation of the rivers with increased flow volumes following the uplifting of the land, which was around 350–400 m inland of the Cape Fold Mountains. Furthermore, the absence of Pleistocene local glaciation events would have allowed a high species survival rate, which over time, provided the opportunity for evolutionary change and diversification among many populations, reflected today by a high ratio of extant genera to species (i.e., many genera with few species) in several aquatic insect groups, as well as in several (but not all, e.g., Ericaceae) fynbos plant families.

These events suggest that the ancient fold mountains provided refuges, allowing species to move along river gradients during times of climatic change when glacial and interglacial periods caused sea recessions and transgressions. Riverine invertebrates likely moved upstream or downstream as they tracked suitable conditions such as thermal refuges at different elevations, while also speciating through isolation of different populations and at different locations along the length of the mountain chain with its huge variety of microclimatic conditions. Furthermore, shifts in seasonal rainfall may have stimulated species to move into more favorable hydrological and thermal conditions as climatic conditions changed.

Furthermore, these movements probably varied according to prevailing rainfall and condensation events, as well as by fire‐protected and forested valleys, screes, kloofs, and riparian zones that are protected by cliffs and scarps. These events were likely repeated many times, resulting in much vicariance and a mosaic of distribution patterns, as well as an adaptation to a winter rainfall‐dry summer regime in the southwest. Additionally, high levels of genetic diversity in the form of many cryptic species have resulted in the formation of species flocks, for example, in the amphipods, isopods, crabs, notonemourid stoneflies, teloganodid mayflies, and leptocerid caddisflies (De Moor & Day, 2013).

Among 38 GCFR dragonfly species surveyed for ectoparasitic mites in Kogelberg Biosphere Reserve, only the widespread generalist species carry mite loads, while the localized endemic dragonfly species are parasite‐free, possibly due to isolation factors inhibiting mite dispersal and colonization and/or an effective immune response (Grant & Samways, 2007a).

**FIGURE 5:**
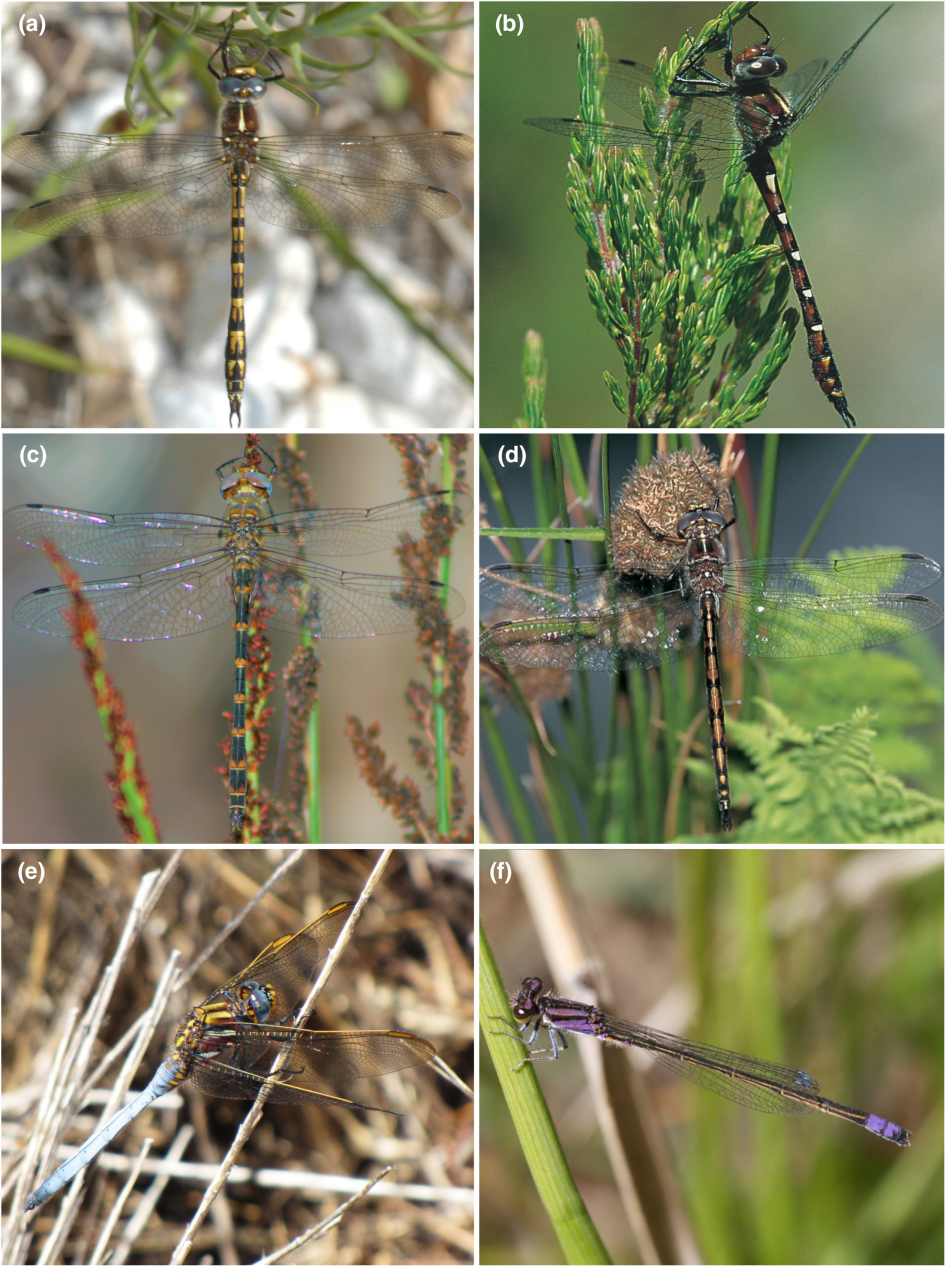
Dragonfly endemism is high in the Greater Cape Floristic Region. Some of the rarest species are *Syncordulia legator* (a), *S. venator* (b), *S. serendipator* (c), *S. gracilis* (d), *Orthetrum rubens* (e), and *Proischnura polychromatica* (f). Photo (e) by K.‐D. Dijkstra.

The GCFR is not especially rich in dragonfly species compared to the wet, northeastern regions of southern Africa. However, Cape Fold Mountains support centers of high local endemism (Deacon et al., 2020), with some of the GCFR endemics having an ancient lineage. The genus *Syncordulia* for example diverged about 60 mya, with three of the four extant species confined to the mountains of the GCFR. *Syncordulia gracilis* is estimated to have appeared at 59.57 mya, *Syncordulia serendipator* 55.34 mya, and *Syncordulia venator* and *Syncordulia legator* 44.85 mya (Ware et al., 2009). All are restricted to specific habitats and locations, while also occurring at low population levels. Other Cape Fold Mountain endemic dragonfly species are in the more widespread genera *Proischnura*, *Pseudagrion*, and *Orthetrum* (Deacon et al., 2020) (Figure 5).

In contrast to the above cases, macroinvertebrates of temporary wetlands in the GCFR consist mainly of widespread and habitat generalist species that colonize these ephemeral habitats (Mlambo et al., 2011), supporting the contention that the water sponge effect of the mountains and perennial stream flows have been important drivers of the high perennial freshwater diversity in the region.

## 6 RELATIONSHIPS BETWEEN PLANTS AND INSECTS

### 6.1 Pollination syndromes

Monkey beetles (Hoplinii) are an important spring pollinator group in the GCFR, with many specialized behaviors (Goldblatt et al., 1998). Overall, the GCFR plant‐pollinator systems show remarkable ecological and evolutionary diversification, especially in the case of the floral rather than vegetative parts of the plants (Johnson, 2010). Sometimes the pollination syndrome is highly specialized, as in the case of the long‐proboscid nemestrinid and tabanid flies, where the long proboscis penetrates the deep cylindrical floral tubes to obtain nectar, especially at specific times of the year. These flies are ecologically significant as the main pollinators of about 200 plant species (Goldblatt & Manning, 2000). In the case of *Megapalpus capensis*, older lineages diverged in Fynbos during the Miocene, while the younger succulent Karoo/Namaqualand lineages diverged during the Pliocene, corresponding with the geological ages of these biomes (de Jager & Ellis, 2017). These flies are strongly associated with their host plants, and these findings present strong evidence for co‐divergence between pollinators and plants in the region. Evidence on the pollination syndrome among *Prosoeca* sp. (Diptera: Nemestrinidae) and host plants also suggest that pollinator traits are determinants of their ecology (Pauw, 2022).

Also associated with the high floral diversity are hotspots with especially high bee diversity in the Fynbos, Succulent Karoo, and Desert biomes, with higher‐than‐expected bee density largely concentrated in Mediterranean‐type and arid habitats (Melin et al., 2024). In addition to supporting many bee species, the GCFR represents one of the global hotspots for bee endemism, especially in the Colletinae subfamily (Bystriakova et al., 2018). However, it is challenging to determine how often co‐speciation events occur, and in the case of oil‐collecting *Rediviva* bees, recent evidence suggests that host plant diversification may be driven by their pollinators, but vice versa, not necessarily (Kahnt et al., 2019).

### 6.2 Levels of congruency between plants and arthropods

There can be similar patterns of endemism between endemic plants and insects, although the relationships are not necessarily causal. For example, on the Cape Peninsula there are about 102 arthropod species that are endemic to this area. They are mostly clustered in forested montane areas, especially on steep slopes. For both plants and arthropod endemics, their congruency appears to be due to similar historical isolation and topography (Picker & Samways, 1996).

Furthermore, the extent to which species turnover among plants and insects are concordant or not depends on the taxa used, the method of insect sampling, and on the spatial scale. Results from insects sweep netted from plants at spatial scales 10 m to 1 km indicated that plant phylogenetic diversity and genera diversity is a better predictor of insect species diversity than plant species diversity. This means that abiotic factors drive both plant and insect diversity, leading to both groups sharing common diversification, immigration, and extinction processes (Procheş et al., 2009).

Despite insect herbivore species richness being comparatively low (Giliomee, 2003), for insect herbivores on Restionaceae plant species, there is strong congruency in species turnover between the insects and plants, likely structured by host specificity. Plant communities show near complete turnover at small spatial scales (0.1–3 km apart), with insects showing a similar pattern, whereas measured environmental and plant structural components have no influence on insect composition (Kemp et al., 2017). Furthermore, insect turnover increases significantly with increasing geographical separation (e.g., among mountains), suggesting an additional effect of biogeographical factors on insect distributions.

In the case of flowering plants and flower‐visiting insects, across different geographic distances (0.5–80 km), there is significant concordance between plants and anthophile assemblages. However, species turnover is weaker in plants than in anthophiles, decreasing with greater geographical distance between plot pairs. In contrast, insect turnover remains high with increasing geographical distance between plot pairs. Furthermore, flowering plant species composition does not have a significant effect on anthophile species composition (Simaika et al., 2018).

### 6.3 Effect of elevation on plant‐anthophile relationships

Elevation also shapes the distribution of arthropods and associated plants in this topographically complex region. As regards species turnover among anthophiles across a 1640 m elevation gradient, there is distinct elevation zoning of flower‐visiting insects. Middle elevations (650–744 m a.s.l) have the highest general species richness and abundance of anthophiles. Interaction frequencies and size of interaction networks are also greatest in the middle zone, as are network diversity, generality, and linkage density, while lowest in the peak zone (nearing 1640 m). The greatest zonal change is between species in the middle compared with the peak zone. Large‐sized monkey beetles, bees, and flies characterize the special assemblage in the peak zone (1576–1640 m a.s.l.). For bees, zonation tracks that of plant assemblages and ambient air temperature is the main driver, with the lowest levels in the peak zone. In contrast, beetle distribution is driven by both flower assemblages and air temperature. In turn, wasp and fly interaction networks are not affected by any of the measured environmental variables. It appears that stresses from increased elevation and reduced temperatures, changing abiotic weather conditions such as strong winds at high elevations, and a decline in flowering plant composition causes a breakdown of interaction networks involving bees and beetles but not that of flies and wasps (Adedoja et al., 2018).

### 6.4 Effect of season on plant–anthophile relationships

In terms of seasonality, species composition and seasonal peaks in abundance among different insect pollinator groups and flowering plants differ over the elevation zones at the same elevation gradient. Bee abundance peaks earlier in the season than the other groups across all elevations. Bee abundance peaks earlier than flowering plants at the middle zone but slightly later than flowering plants at the lowest elevations, suggesting a mismatch. This indicates that while elevation shapes species distribution, it also differentially influences species phenology, which may be of significance in these times of rapid climate change in these sensitive mountain ecosystems (Adedoja et al., 2020).

**FIGURE 6:**
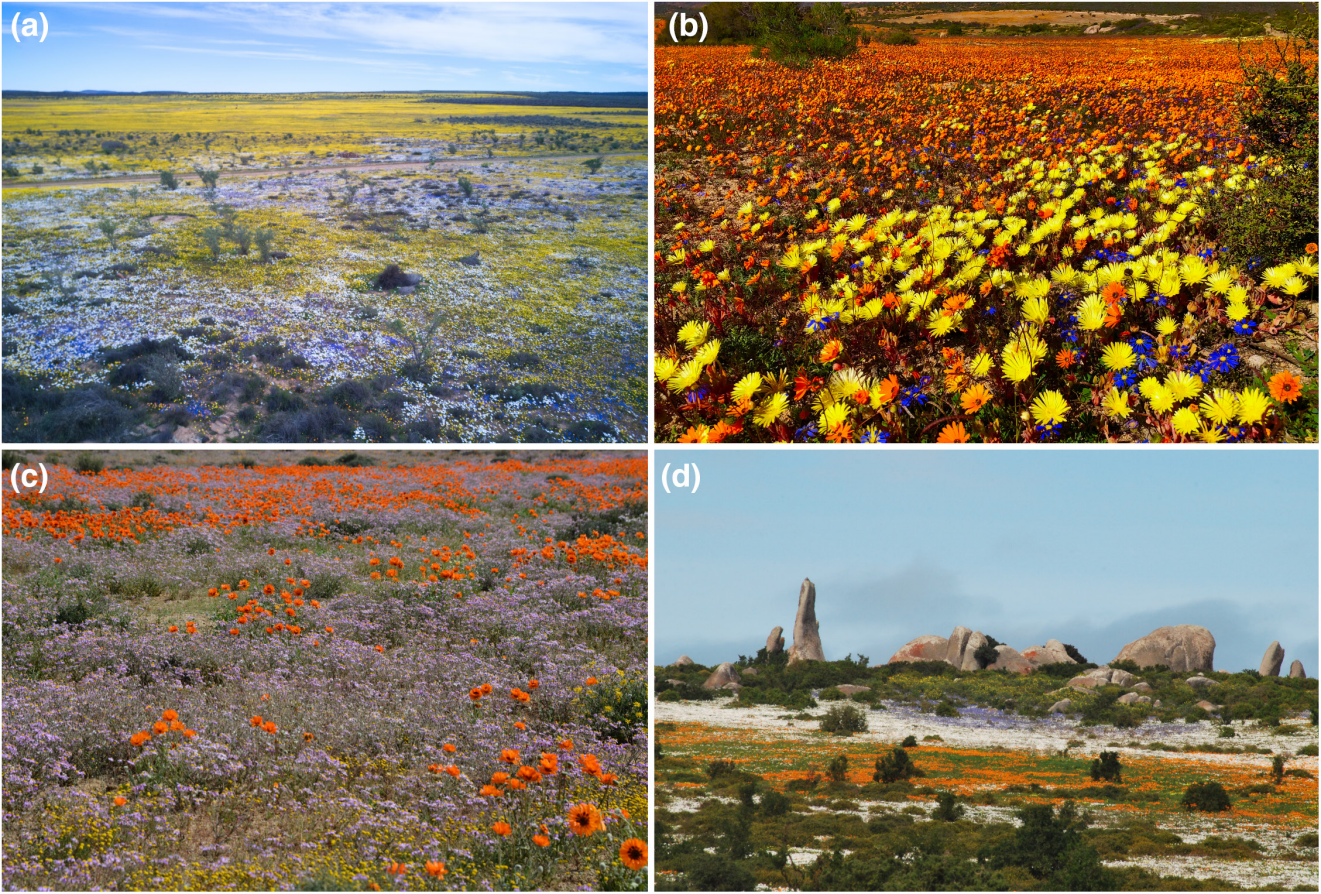
In the Namaqualand Region, in particular, mass flowering events follow high winter rainfall. Aerial views (a) showcase dense flower carpets stretching across the landscape. Color mosaics are variable among flowering events and geographic areas (b–d).

A dramatic feature of the GCFR is mass flowering events, where flowering plants carpet the ground in a mosaic of different color patches. These events come about from a “magnet effect” where anthophile visitation rates and diversity are positively affected by floral density, diversity, and community structure. It appears that intense selection pressure for mass flowering has stimulated the evolution of these spectacular events (Vrdoljak et al., 2016) (Figure 6).

### 6.5 Other close relationships between plants and insects

Aizoaceae plants are a major floral component of the GCFR. This diverse group of succulent plants also supports a high diversity of gall‐forming midge species (Cecidomyiidae), many of which were only recently discovered through molecular work (Dorchin et al., 2022). In the case of the intimate relationship between gall‐insect species and woody shrubs in the GCFR, plant species diversity generates insect species diversity (Wright & Samways, 1996), with high levels of local endemism in both groups (Wright & Samways, 1998). Furthermore, the fynbos Proteaceae has very few borer species in common with those associated with Proteaceae in the northern parts of South Africa, with each plant biogeographical group having its own set of distinct species (Wright & Samways, 2000).

**FIGURE 7:**
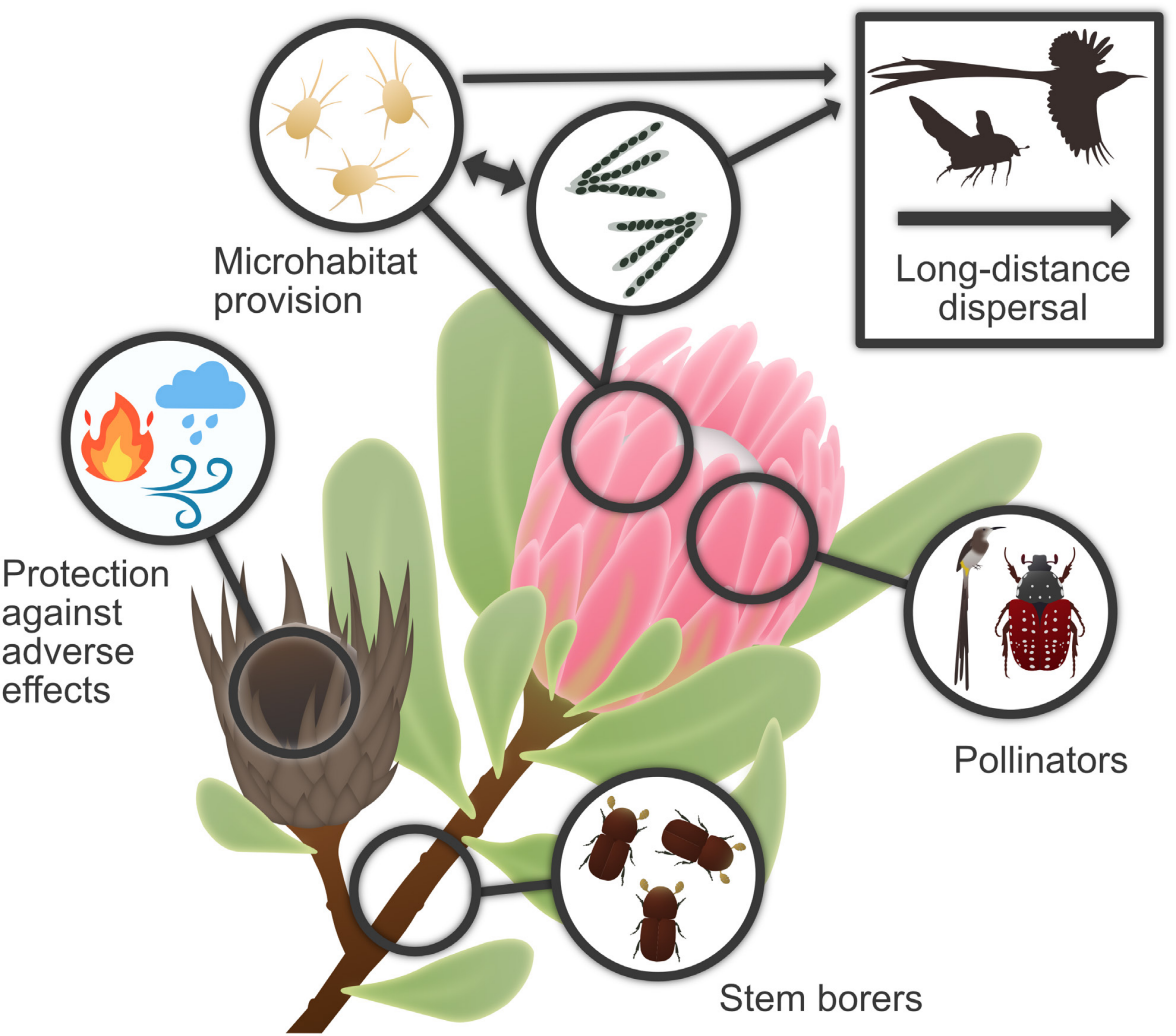
Proteaceae infructescences support a range of multispecies interactions. Borer species take advantage of exposed stems, and individual flowers that make up infructescences are pollinated by birds and beetles. Infructescences provide microhabitats to mites and fungi and rely on pollinating species for long‐distance dispersal. Even dried‐out infructescences from previous flowering seasons are used by a range of species as a refuge from adverse environmental conditions (based on Aylward et al., 2023).

Some intriguing ecological interactions among endemic symbionts take place on the large *Protea* flower heads (infructescences) in the GCFR. These infructescences, which can remain on the plant for several years, are microbiomes that offer protection from weather events and fire even after the flowering period. While the flowers are pollinated by *Genuchus hottentottus* and two *Trichostetha* spp., as well as the Orange‐breasted sunbird (*Nectarinia violacea*) and Cape sugarbird (*Promerops cafer*), five species of mites (*Tarsonemus* spp., *Trichouropoda* sp., *Proctolaelaps vandenbergi*, and tentatively a *Glycyphagus* sp.), several ophiostomatoid fungi, and bacteria continually inhabit the infructescences. The fungi depend on mites for spore dissemination, while the mites attach to pollinating beetles and birds for long‐distance dispersal (reviewed by Aylward et al., 2023) (Figure 7).

## 7 FIRE AS A MAJOR HISTORICAL DRIVER OF LOCAL BIODIVERSITY

Soil type, local climate, and fire are among the major determinants of several biome types in the GCFR, with forests the exception as they largely escape fire impacts (Cowling & Potts, 2015). Regarding plant survival at the community level, species presence and abundance are stable at the metacommunity scale over a 30‐year period but highly unstable at the local scale and are not influenced by species' biological attributes. Overall, stochastic environmental fluctuations associated with recurrent fire buffer plant populations from extinction. This appears to allow niche differentiation which ensures stable coexistence at the meta‐community scale (Thuiller et al., 2007).

The relationship between fire and arthropods has been honed by many millennia of fire events, and is highly complex, with extant species having many adaptations for surviving the intense natural selective force of fire (Janion‐Scheepers, Measey, et al., 2016). However, there is much differential resilience among the various taxa and guilds for surviving fire.

Yekwayo et al. (2018) showed that comparing 3 months, 1 year, and 7 years post‐fire, the response of surface‐active arthropods varies greatly among taxa, with all time‐since‐fire categories supporting distinctive assemblages. This suggests that fire is adding to the landscape heterogeneity and promoting biodiversity as different taxa are using the same area, but at different times since the fire. Some species are present across all temporal categories, while others are present only in a single time‐since‐fire category.

The three proposed strategies for arthropod species to survive are (1) to flee ahead of the fire, only available to very mobile taxa; (2) find refuge, with many of the GCFR taxa using rocky areas with recesses, or burrow in soil, among roots, or occupy termite mounds; or (3) recolonize from unburned areas (New, 2014). All three strategies are evident in the GCFR as some arthropod groups are locally highly resilient to fire (especially ants that move underground), while others are locally less tolerant (dragonflies and pollinators that move to fire refuges), while large grasshoppers fly away from the fire front.

**FIGURE 8:**
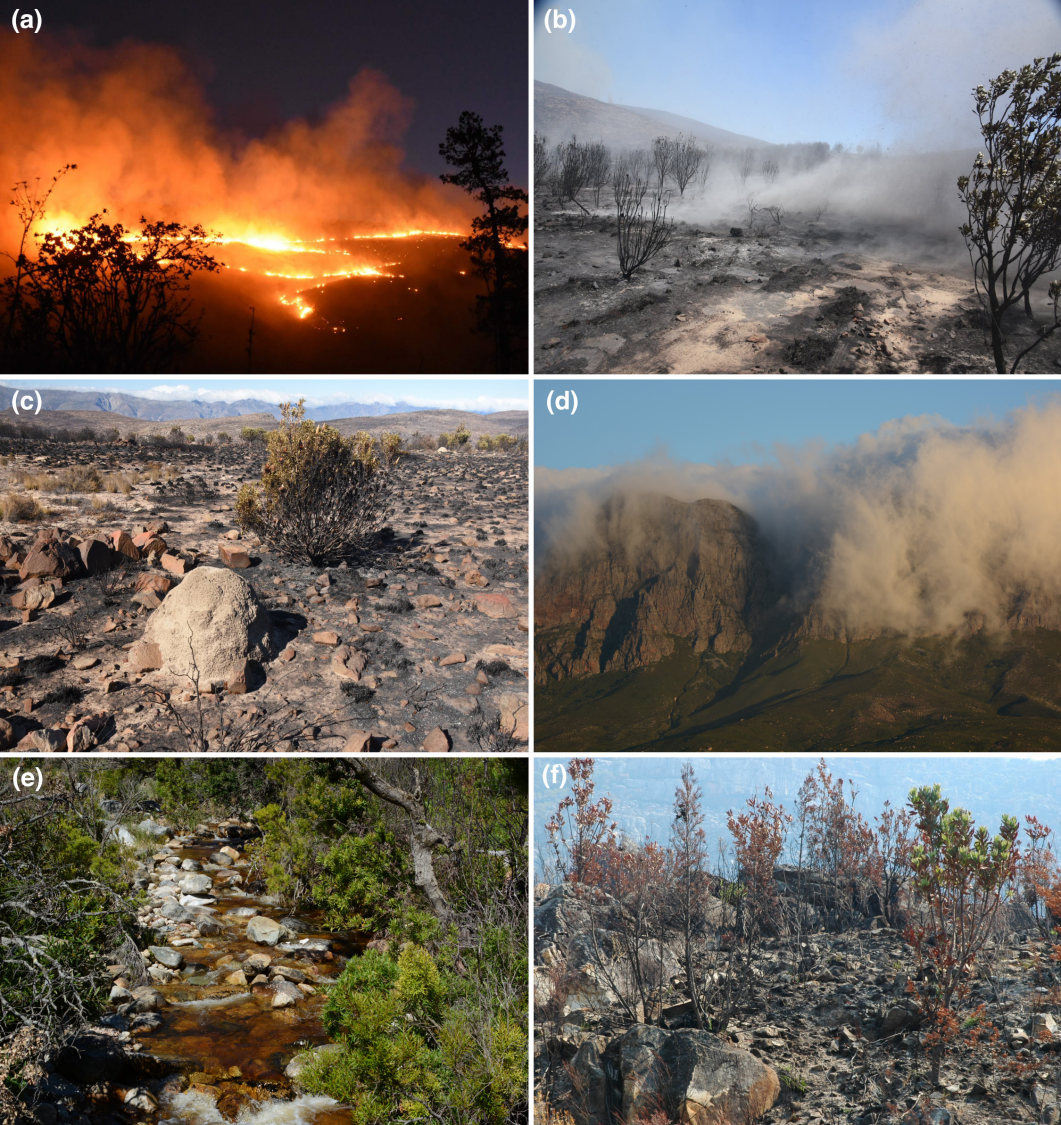
Fire (a) and smoke (b) are major ecological components of the Greater Cape Floristic Region, leading to barren landscapes with high surface ash loads (c). Mountain valleys (d) and riparian areas (e) are important refuges, while recently burned patches (f) are important foraging areas for a range of arthropod species.

Endemic dragonflies (Odonata) are fire‐adapted in the GCFR. With the onset of fire and its attendant smoke plumes, the adults immediately retreat to the riparian zones at the river's edge where they lie low among the marginal vegetation and large boulders until the fire has passed (Samways & Deacon, 2022). The fire‐resilient groups use patch and landscape features as fire refuges (Figure 8). Rockiness affects species richness, abundance, and composition of some taxa, which use this coarse terrain to escape fire (Pryke & Samways, 2012). Some fire‐resilient groups, for example, ants emerge from soil burrows and then take advantage of the newly burned areas.

**FIGURE 9:**
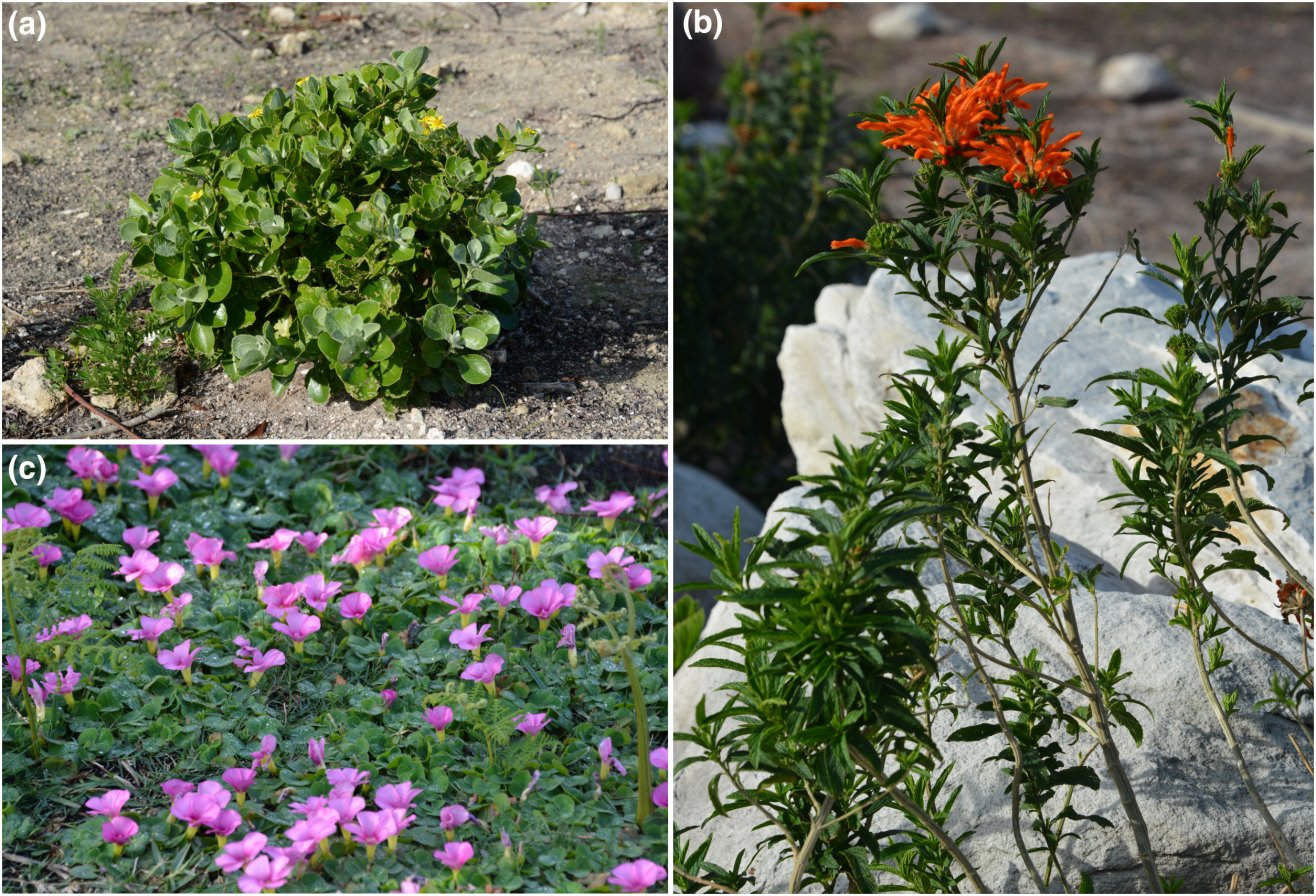
Many flowering plant species recover within 6 months post‐fire. Regrowing plants bloom on an otherwise ash‐covered background (a and b), with some soon covering large patches in the landscape (c).

The patchy nature of the burns in GCFR leaves many small areas unburned, allowing species to recolonize from nearby unburned areas (Janion‐Scheepers, Measey, et al., 2016; Yekwayo et al., 2018). Pollinators are absent immediately after a fire but have their highest activity levels 3–6 months after fynbos burns. Many fynbos plants flower shortly after the fire, and this attracts pollinators from the unburned refuges (Adedoja et al., 2019; Pryke & Samways, 2012) (Figure 9). These responses indicate that characteristics of local landscape structure, including having refuges and landscape connectivity, as well as frequency and intensity of fire, are historical drivers of the local distribution of arthropods in the GCFR.

Although some arthropod species are vulnerable at the local scale, few species are vulnerable at the landscape scale, having historically evolved with fire. Furthermore, many fynbos insects rely on fires to maintain suitable habitat structures and plant diversity (Topp et al., 2021). The notable exception here are two lycaenid butterflies (the scarce mountain copper *Trimenia malagrida malagrida* and the Brenton blue *Orachrysops niobe*), for which no living individuals have been recorded since intense fires burned their last known areas of extent, in 1994 and 2017, respectively.

In sum, the relationships between fire and arthropods are phylogenetically constrained, having been honed by many millennia of fire events, and are highly complex. Present‐day species manifest a variety of adaptations for escaping the great natural selective force of fire, whether through movement to fire refugia, or through varied expression of life‐history traits such as diurnal versus nocturnal activity and/or location of immature stages relative to plant tissue (JaVale et al., 2023).

## 8 EL NIÑO SOUTHERN OSCILLATION EVENTS AND WINTER RAINFALL AS DRIVERS OF ARTHROPOD DIVERSITY

The GCFR, and most of southern‐eastern Africa, is subject to strong El Niño Southern Oscillation (ENSO) events, leading to pronounced wet‐dry cycles (Gagan, 2009), which have resulted in alternating drought and fire cycles over the last 12,000 years. Many species have adapted to these events and to opportunities provided by humans. For example, some localized lotic dragonfly species (*Pseudagrion draconis*, *Pseudagrion furcigerum*, *Zosteraeschna minuscula*, *Pinheyschna subpupillata*, and *Syncordulia venator*) during drought years switch to low‐quality lentic environments (artificial water bodies) to survive droughts. Perhaps prior to human intervention, they temporarily used other sub‐optimal habitats, such as deposition pools (Deacon et al., 2019).

Interestingly, GCFR arthropod species have not yet been observed showing significant shifts in geographic ranges due to climate change, as in the Northern Hemisphere. This might possibly be attributed to ENSO, with species having survived a variety of dry‐wet climatic cycles for millennia.

As winter rainfall prevails in this region, the phenology of the GCFR species is different from the rest of South Africa, with peaks in adult emergence occurring for different groups at different times of the year. For example, many bees and other pollinators emerge in early spring as most flowers are present during this period, while many butterflies and beetles emerge in the late spring to take advantage of the last rains and the early warm periods. Moths and widows (Satyrinae butterflies) emerge in autumn (the first rains of the year while there is still some summer heat) and dung beetles have their highest activity in winter (the wettest part of the year). In the GCFR, fewer species emerge and fly in mid‐summer than do in spring or autumn (Pryke & Samways, 2008; Samways & Grant, 2007), most likely to avoid the hot dry summers with stressful environments and associated fires, especially during dry ENSO cycles. The advantage of strong adaptation to these conditions is seen among dragonflies in the Kogelberg Biosphere Reserve, where 53% of dragonfly individuals and 26% of species were GCFR or national endemics (Grant & Samways, 2007b).

## 9 THE CAPE PENINSULA AND ITS ICONIC TABLE MOUNTAIN

**FIGURE 10:**
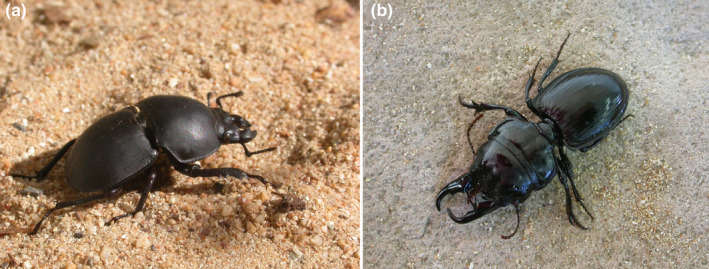
*Colophon westwoodi* (a) and *Pachyodontus languidus* (b), both endemic to Table Mountain, South Africa.

Within the GCFR there are some areas that stand out for their unique biodiversity, with the Cape Peninsula having 158 endemic angiosperm species in an area of only 470 km^2^ (Helmet & Trinder‐Smith, 2006). To assess whether the fauna had similar levels of endemism to plants, Picker and Samways (1996) reviewed the Cape Peninsula and recorded 112 endemic faunal species, of which 111 were invertebrates. This included the charismatic *Colophon westwoodi* and the distinctive carabid (*Pachyodontus languidus*) (Figure 10). Since then, the Cape Peninsula has also been identified as a national center of endemism for both Onychophora (Hamer et al., 1997) and Diplopoda (Hamer & Slotow, 2002).

Turner (2006) sampled water beetles on the top of Table Mountain and showed that 77% of all water beetles on Table Mountain were endemic to the Cape Floristic Region component of the GCFR. Furthermore, there are five red‐listed butterflies on the peninsula, two of which are Cape Peninsula endemics (Mecenero et al., 2013).

Many new species have also been described from the Cape Peninsula since this assessment, including the world's only known jumping cockroach (*Saltoblattella montistabularis*) (Picker et al., 2011) and a phylogenetically unique diving beetle (*Capelatus prykei*), with no known relatives in Africa (Bilton et al., 2015). The Cape Peninsula, like the Kogelberg Biosphere Reserve (Grant & Samways, 2011), is a hotspot within a biodiversity hotspot and is one of the most unique biological areas in the world. For instance, Table Mountain supports a high density of Red‐Listed insect species, while Cape Point, the southernmost tip of the peninsula, supports species found nowhere else on the peninsula (Pryke & Samways, 2009). Despite the Cape Peninsula being one of the most sampled areas in South Africa, new and phylogenetically interesting species are still being discovered, highlighting how little we know of entomofauna of much of the GCFR.

**FIGURE 11:**
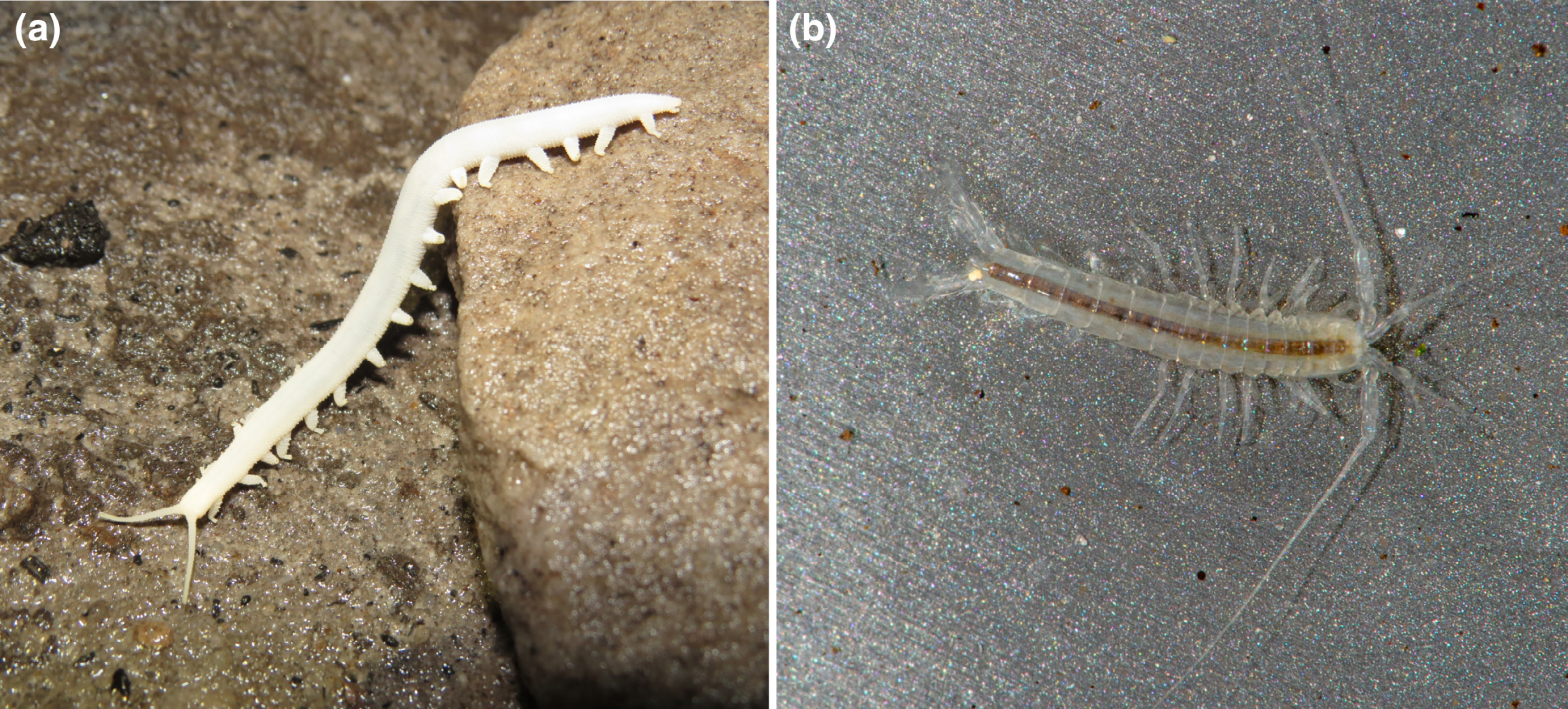
*Peripatopsis alba* (a) and *Spelaeogriphus lepidops* (b), both troglobitic endemics to the cave systems on the Cape Peninsula, South Africa. Photos by Rodrigo Lopes Ferreira (a) and Gonzalo Giribet (b).

The deep cave system inside Table Mountain is of ancient phreatic origin in the hard quartzitic sandstone (Marker & Swart, 1995). The system supports several troglobitic endemics including the white‐colored onychophoran *Peripatopsis alba* and the biogeographically isolated crustacean *Spelaeogriphus lepidops* (Sharratt et al., 2000) (Figure 11). It also supports the world's first discovered fully troglobitic and highly modified Gymnobisiidae (Pseudoscorpiones: Neobisioidea) species (Harvey et al., 2016). A total of 18 troglobitic arthropod species have been recorded to date, making it a hotspot of cave biodiversity, with all but one being arthropods (Ferreira et al., 2020). However, as the huge labyrinth of caves and cracks is mostly inaccessible to humans, more endemic species are likely to be present.

## 10 CONSERVATION IMPLICATIONS

Effective conservation depends, among many things, on a sound base of historical biogeography. Not only does this base provide insight into aspects like the extent of occurrence and area of occupancy but also on other spatial aspects such as beta and gamma diversity. Furthermore, it provides considerable information on ecological preferences, species interactions, and which are historic drivers as opposed to novel ones from human impact.

A critically important early driver of plant and arthropod evolution has been the extremely old Cape Fold Mountains, the peaks of which are 8–10 times older than many of the major ranges around the world that arose in the Cenozoic. This ancient and complex topography, coupled with the lack of any glacial blankets for over 200 my set the stage for much evolutionary diversification. This stage then experienced crustal, climatic, and sea level change through the Cenozoic, but nevertheless retained a multitude of refugia, allowing evolutionary vicariance to take place.

Overall, the arthropod fauna, like the flora, is of cosmopolitan origin. The GCFR has been a cradle of ancient plant and arthropod diversity, retaining many early lineages but has also received early arrivals from farther north. Then, since the PETM, there has been considerable speciation in many groups. This speciation is continuing today as shown by the many new cryptic arthropod species being discovered.

Against this background, the crux of arthropod conservation, like that of plants on which many species depend, is knowing where these species occur. This means that much more exploration needs to take place. This includes not only just finding new species and localities but also establishing which interspecific interactions are important. In short, conservation activities in the GCFR must inevitably be highly area specific.

Of greatest concern for both plants and arthropods are the GCFR lowlands where human pressure is greater than the better‐protected mountains. Above all, this means more effective agroecology and town planning.

A final note is that while narrow‐range endemics may appear highly susceptible, this is a spatially explicit issue. After all, the species survivors today are the ones who overcame many past environmental filters. One of the greatest impacts on both plants and arthropods is from invasive alien plants. But when these are removed, rare, ancient, endemic dragonfly populations, for example, can bounce back remarkably well (Samways & Sharratt, 2010). Details of the various arthropod conservation strategies will be discussed elsewhere.

## 11 CONCLUSIONS

Overall, it has been the variety of deep and more recent geological, topographical, hydrological, and ecological factors that have driven both arthropod diversity and local distribution and persistence of certain arthropod lineages in the GCFR in the long term. The effects of these past drivers were both direct on arthropod populations and mediated by plants and the food chains supported by these primary producers. During the last 65 my, many new lineages appeared, leading to much disparity along with considerable later speciation in many genera. Many of these species have persisted until modern times.

The Pleistocene saw many new major events in response to worldwide effects of glacial advances and retreats. Although the GCFR was not glaciated, sea regressions and transgressions were significant for the region. Of special note was the appearance of the Palaeo‐Agulhas Plain, with its highly productive ecosystems, which today is submerged. However, these sea‐level changes drove population shifts, vicariance, and more recent speciation, resulting in many cryptic species complexes.

Interestingly, increasingly high numbers of species and molecular taxonomic operational units have similarly diversified, whether terrestrial, aquatic, or cavernicolous. Mostly this diversification has resulted in high levels of beta and gamma diversity, but not necessarily high levels of alpha diversity. However, today some lineages are sympatric, as suitable habitats have expanded, such as onychophorans in forests. Not only has habitat specialization been a feature of GCFR arthropods but also has many changes in specialized morphologies and species interactions, ranging from long‐proboscid flies feeding on long corolla flowers, orthopterans associated with specific plant species, to complex interactive microbiomes inside *Protea* flower heads.

New GCFR endemic species are being regularly discovered in many taxonomic and functional groups, especially through targeted field searches and molecular investigation. These advances are likely to further increase knowledge of GCFR megadiversity.

The take home message arising from this eco‐evolutionary perspective is that specific regional past geological events and climates have had a huge influence on the high diversity and spatial patterning of both terrestrial and aquatic biota in the GCFR, qualifying the region as a megadiversity hotspot. Furthermore, the diversity and spatial patterns of many terrestrial plant species have been highly influential in shaping arthropod diversity and species turnover, with both plants and arthropods displaying high levels of local endemism. These findings emphasize the importance of formulating conservation strategies that recognize the strong influence of past environmental filters and present conditions on both terrestrial and aquatic arthropods. In addition, as plant and arthropod conservation are intimately related and with the presence of so many irreplaceable species, fine‐grain spatial conservation strategies are essential and instigated over large areas to encompass all the arthropod diversity.

## AUTHOR CONTRIBUTIONS

**Michael J. Samways:** Conceptualization (equal); investigation (equal); visualization (equal); writing – original draft (equal); writing – review and editing (equal). **James S. Pryke:** Conceptualization (equal); investigation (equal); visualization (equal); writing – original draft (equal); writing – review and editing (equal). **René Gaigher:** Conceptualization (equal); investigation (equal); visualization (equal); writing – original draft (equal); writing – review and editing (equal). **Charl Deacon:** Conceptualization (equal); investigation (equal); visualization (equal); writing – original draft (equal); writing – review and editing (equal).

## FUNDING INFORMATION

The National Research Foundation, Stellenbosch University, and Mondi Group provided funding for this work.

## CONFLICT OF INTEREST STATEMENT

The authors have no conflicts of interest to declare.

## Data Availability

No primary data were collected or analysed during the preparation of this review manuscript.
